# The *R2R3-MYB* gene family in banana *(Musa acuminata)*: Genome-wide identification, classification and expression patterns

**DOI:** 10.1371/journal.pone.0239275

**Published:** 2020-10-06

**Authors:** Boas Pucker, Ashutosh Pandey, Bernd Weisshaar, Ralf Stracke

**Affiliations:** 1 Faculty of Biology, Genetics and Genomics of Plants, Bielefeld University, Bielefeld, Germany; 2 National Institute of Plant Genome Research, New Delhi, India; Aberystwyth University, UNITED KINGDOM

## Abstract

The *R2R3-MYB* genes comprise one of the largest transcription factor gene families in plants, playing regulatory roles in plant-specific developmental processes, defense responses and metabolite accumulation. To date MYB family genes have not yet been comprehensively identified in the major staple fruit crop banana. In this study, we present a comprehensive, genome-wide analysis of the *MYB* genes from *Musa acuminata* DH-Pahang (A genome). A total of 285 *R2R3-MYB* genes as well as genes encoding three other classes of MYB proteins containing multiple MYB repeats were identified and characterised with respect to structure and chromosomal organisation. Organ- and development-specific expression patterns were determined from RNA-Seq data. For 280 *M*. *acuminata MYB* genes for which expression was found in at least one of the analysed samples, a variety of expression patterns were detected. The *M*. *acuminata R2R3-MYB* genes were functionally categorised, leading to the identification of seven clades containing only *M*. *acuminata* R2R3-MYBs. The encoded proteins may have specialised functions that were acquired or expanded in *Musa* during genome evolution. This functional classification and expression analysis of the *MYB* gene family in banana establishes a solid foundation for future comprehensive functional analysis of MaMYBs and can be utilized in banana improvement programmes.

## Introduction

Banana (*Musa* spp.), including dessert and cooking types, is a staple fruit crop for a major world population, especially in developing countries. The crop is grown in more than 100 countries throughout the tropics and sub-tropics, mainly in the African, Asia-Pacific, and Latin American and Caribbean regions [1]. Bananas provide an excellent source of energy and are rich in certain minerals and in vitamins A, C and B6. Furthermore, this perennial, monocotyledonous plant provides an important source of fibre, sugar, starch and cellulose (used for paper, textiles). Bananas have also been considered as a useful tool to deliver edible vaccines [2]. Certain agronomic traits, such as stress and pest resistance as well as fruit quality, are thus of considerable interest. Banana improvement through breeding exercises has been challenging for various reasons. Therefore, genetic engineering-based optimisations hold great promise for crop improvement. For this purpose, candidate gene targets need to be identified. The release of a high quality banana genome sequence [3] provides an useful resource to understand functional genomics of important agronomic traits and to identify candidate genes to be utilized in banana improvement programmes.

Almost all biological processes in eukaryotic cells or organisms are influenced by transcriptional control of gene expression. Thus, the regulatory level is a good starting point for genetic engineering. Regulatory proteins are involved in transcriptional control, alone or complexed with other proteins, by activating or repressing (or both) the recruitment of RNA polymerase to promoters of specific genes [4]. These proteins are called transcription factors. As expected from their substantial regulatory complexity, transcription factors are numerous and diverse [5]. By binding to specific DNA sequence motifs and regulating gene expression, transcription factors control various regulatory and signaling networks involved in the development, growth and stress response in an organism.

One of the widest distributed transcription factor families in all eukaryotes is the MYB (myeloblastosis) protein family. In the plant kingdom, MYB proteins constitute one of the largest transcription factor families. MYB proteins are defined by a highly conserved MYB DNA-binding domain, mostly located at the N-terminus of the protein. The MYB domain generally consists of up to four imperfect amino acid sequence repeats (R) of about 50–53 amino acids, each forming three alpha–helices [summarised in 6]. The second and third helices of each repeat build a helix–turn–helix (HTH) structure with three regularly spaced tryptophan (or hydrophobic) residues, forming a hydrophobic core [7]. The third helix of each repeat was identified as the DNA recognition helix that makes direct contact with DNA [8]. During DNA contact, two MYB repeats are closely packed in the major groove, so that the two recognition helices bind cooperatively to the specific DNA recognition sequence motif. In contrast to vertebrates genomes, which only encode MYB transcription factors with three repeats, plants have different MYB domain organisations, comprising one to four repeats [6, 9]. R2R3-MYBs, which are MYB proteins with two repeats (named according to repeat numbering in vertebrate MYBs), are particularly expanded in plant genomes. Copy numbers range from 45 unique *R2R3-MYBs* in *Ginkgo biloba* [10] to 360 in Mexican cotton (*Gossypium hirsutum*) [11]. The expansion of the R2R3-MYB family was coupled with widening in the functional diversity of R2R3-MYBs, considered to regulate mainly plant-specific processes including secondary metabolism, stress responses and development [6]. As expected, R2R3-MYBs are involved in regulating several biological traits, for example wine quality, fruit color, cotton fibre length, pollinator preferences and nodulation in legumes.

In this study, we have used genomic resources to systematically identify members of the *M*. *acuminata* (A genome) *R2R3-MYB* gene family. We used knowledge from other plant species, including the model plant *A*. *thaliana*, leading to a functional classification of the banana *R2R3-MYB* genes based on the MYB phylogeny. Furthermore, RNA-Seq data was used to analyse expression in different *M*. *acuminata* organs and developmental stages and to compare expression patterns of closely grouped co-orthologs. The identification and functional characterization of the *R2R3-MYB* gene family from banana will provide an insight into the regulatory aspects of different biochemical and physiological processes, as those operating during fruit ripening as well as response to various environmental stresses. Our findings offer the first step towards further investigations on the biological and molecular functions of MYB transcription factors with the selection of genes responsible for economically important traits in *Musa*, which can be utilized in banana improvement programmes.

## Material and methods

### Search for MYB protein coding genes in the *M*. *acuminata* genome

A consensus R2R3-MYB DNA binding domain sequence [12] (S1 Table) was used as protein query in tBLASTn [13] searches on the *M*. *acuminata* DH-Pahang genome sequence (version 2) (https://banana-genome-hub.southgreen.fr/sites/banana-genome-hub.southgreen.fr/files/data/fasta/version2/musa_acuminata_v2_pseudochromosome.fna) in an initial search for MYB protein coding genes. To confirm the obtained amino acid sequences, the putative MYB sequences were manually analysed for the presence of an intact MYB domain. All *M*. *acuminata* MYB candidates from the initial BLAST were inspected to ensure that the putative gene models encode two or more (multiple) MYB repeats. The identified gene models were analysed to map them individually to unique loci in the genome and redundant sequences were discarded from the data set to obtain unique *MaMYB* genes. The identified *MaMYB* genes were matched to the automatically annotated genes from the Banana Genome Hub database [14]. The open reading frames of the identified *MaMYB* were manually inspected and, if available, verified by mapping of RNA-Seq data to the genomic sequence. Resulting *MaMYB* annotation is provided in the supporting information: Multi-FASTA files with *MaMYB* CDS sequences (S1 File) and MaMYB peptide sequences (S2 File), general feature format (GFF) file (S3 File) describing the *MaMYB* genes for use in genome viewers/browers.

### Genomic distribution of *MaMYB* genes

The *MaMYB* genes were located on the corresponding chromosomes by the MapGene2chromosome web v2 (MG2C) software tool (http://mg2c.iask.in/mg2c_v2.0/) according to their position information of a physical map, available from the DH-Pahang genome annotation (V2).

### Phylogenetic analyses

Protein sequences of 133 *A*. *thaliana* MYBs were obtained from TAIR (http://www.arabidopsis.org/). We also considered other multiple MYB-repeat proteins from *A*. *thaliana* in the phylogenetic analysis to determine orthologs in the *M*. *acuminata* genome: five AtMYB3R, AtMYB4R1 and AtCDC5. Additionally, 43 well-known, functionally characterised landmark plant R2R3-MYB protein sequences were collected from GenBank at the National Center for Biotechnology Information (NCBI) (http://www.ncbi.nlm.nih.gov/).

Phylogenetic trees were constructed from ClustalΩ [15] aligned MYB domain sequences (293 MaMYBs, 132 AtMYBs and 43 plant landmark MYBs) using MEGA7 [16] with default settings. A mojority rule Maximum Likelihood (ML) consensus tree inferred from 1000 bootstrap replications was calculated. *M*. *acuminata* MYB proteins were classified according to their relationships with corresponding *A*. *thaliana* and landmark MYB proteins.

### Expression analysis from RNA-Seq data

RNA-Seq data sets were retrieved from the Sequence Read Archive (https://www.ncbi.nlm.nih.gov/sra) via fastq-dump v2.9.6 (https://github.com/ncbi/sra-tools) (S2 Table). STAR v2.5.1b [17] was applied for the mapping of reads to the Pahang v2 reference genome sequence [18] using previously described parameters [19]. featureCounts [20] was applied for quantification of the mapped reads per gene based on the Pahang v2 annotation. Previously developed Python scripts were deployed for the calculation of normalized expression values (https://github.com/bpucker/bananaMYB) [19].

## Results and discussion

The first annotated reference genome sequence of *M*. *acuminata* (A genome) became available in 2012 [3]. It was obtained from a double haploid (DH) plant of the Pahang cultivar, derived through haploid pollen and spontaneous chromosome doubling from the wild subspecies *Musa acuminata* ssp. *malaccensis* [3]. This wild subspecies was involved in the domestication of the vast majority of cultivated bananas and its genetic signature is commonly found in dessert and cooking bananas (ProMusa, http://www.promusa.org). An improved version (DH-Pahang version 2) of the genome assembly and annotation was presented in 2016 comprising 450.7 Mb (86% of the estimated size) from which 89.5% are assigned to one of the 11 chromosomes, predicted to contain 35,276 protein encoding genes [18]. This *M*. *acuminata* DH-Pahang version 2 genome sequence provides the platform of this study.

### Identification and genomic distribution of *M*. *acuminata MYB* genes

A consensus R2R3-MYB DNA binding domain sequence (deduced from Arabidopsis, grape and sugarbeet R2R3-MYBs, S1 Table) was used as protein query in tBLASTn searches on the DH-Pahang version 2 genome sequence to comprehensively identify MYB protein coding genes in *M*. *acuminata*. The resulting putative MYB sequences were proven to map to unique loci in the genome and were confirmed to contain an intact MYB domain. This ensured that the gene models contained two or more (multiple) MYB repeats. We identified a set of 285 R2R3-MYB proteins and nine multiple repeat MYB proteins distantly related to the typical R2R3-MYB proteins: six R1R2R3-MYB (MYB3R) proteins, two MYB4R proteins and one CDC5-like protein from the *M*. *acuminata* genome sequence (Table 1).

**Table 1:** List of annotated *MYB* genes in the *Musa acuminata* (DH Pahang) genome sequence.

		*pseudochr*. *position*			*BHLH*	*coding*	*peptide*	*MYB *	* *
*gene ID*	*synonym*	*from*	*to*	*str*.	*motif*	*exons*	*length*	*type*	*functional assignment*
Ma01_g00440		314253	315377	-		4	297	R2R3	stress response, hormone signaling
Ma01_g02850	MusaMYB31 [21]	1866501	1867245	+	+	3	200	R2R3	repressors PP, sinapate, lignin
Ma01_g04470		3005032	3006551	+		4	230	R2R3	ASR, flower morphogenesis, stilbene
Ma01_g10440		7520585	7526901	+		3	556	R2R3	anther development, stress response
Ma01_g10750		7722708	7723839	-		3	273	R2R3	photomorphogenesis
Ma01_g11890		8618982	8620082	+		2	234	R2R3	secondary cell wall, lignin
Ma01_g12250		8870030	8871190	-		3	334	R2R3	
Ma01_g14370		10499811	10500874	+		4	195	R2R3	defense, stress response
Ma01_g15800		11477487	11478908	+		4	197	R2R3	secondary cell wall, lignin
Ma01_g16960		12409268	12410289	+		3	293	R2R3	
Ma01_g17260		12621891	12623358	-		5	360	R2R3	
Ma01_g17450		12782371	12783358	+		3	270	R2R3	defense, stress response
Ma01_g18470		13704465	13705557	-		2	330	R2R3	suberin
Ma01_g19610	MYB32 [22]	15271319	15272110	-	+	2	241	R2R3	repressors PP, sinapate, lignin
Ma01_g19960		15935305	15936912	-		5	282	R2R3	stress response, hormone signaling
Ma01_g21340		20977298	20978421	-		3	196	R2R3	stamen development
Ma02_g00280		2702046	2703056	+		3	281	R2R3	defense, stress response
Ma02_g00290		4086487	4089065	+		3	320	R2R3	flavonols, phlobaphene
Ma02_g03780		15236983	15238054	+		3	307	R2R3	mucillage, lignin, stomatal closure
Ma02_g04860		16268865	16270029	+		3	325	R2R3	
Ma02_g05880		17024294	17029382	-		7	489	3R	cell cycle control
Ma02_g06190		17225149	17226252	+		3	301	R2R3	axillary meristem, root growth
Ma02_g06670		17595551	17596584	+		2	317	R2R3	suberin
Ma02_g09720	MYB46 [22]	19581503	19582518	+		2	297	R2R3	secondary wall, lignin
Ma02_g09870		19645550	19646834	-		3	381	R2R3	
Ma02_g13370		21804465	21806215	+		3	280	R2R3	photomorphogenesis
Ma02_g15770		23338515	23340804	-		3	444	R2R3	anther development, stress response
Ma02_g16570		23869343	23870389	+		3	297	R2R3	root development
Ma02_g17950	MYB48 [22]	24668376	24669235	-		4	205	R2R3	
Ma02_g19650		25862380	25864514	+	+	3	258	R2R3	
Ma02_g19770	MYB63 [22]	25962707	25963911	-		4	303	R2R3	PP, lignin
Ma02_g20270		26305481	26306910	+		4	365	R2R3	
Ma02_g21230		26925219	26926055	+		2	244	R2R3	general flavonoid, trichome
Ma02_g21760		27304216	27317415	-		11	1076	3R	cell cycle control
Ma02_g22540		27850957	27852253	+		3	229	R2R3	repressors PP, sinapate, lignin
Ma02_g23870		28706512	28707545	+		4	253	R2R3	defense, stress response
Ma02_g24520		29095026	29096192	-		3	292	R2R3	defense, stress response
Ma03_g01260		944774	947014	+		4	124	R2R3	defense, stress response
Ma03_g06410		4438262	4439441	-		4	287	R2R3	root development
Ma03_g07620		5357989	5358809	-		3	205	R2R3	
Ma03_g07840 *		5566137	5567139	-	+	3	282	R2R3	proanthocyanidins
Ma03_g07850 *		5573753	5574630	+	+	3	238	R2R3	proanthocyanidins
Ma03_g08300		5982225	5985925	-		5	287	R2R3	defense, stress response
Ma03_g08930		6570843	6573019	+		3	360	R2R3	embryogenesis, seed maturation
Ma03_g09310		6861304	6862367	+		3	289	R2R3	defense, stress response
Ma03_g09340		6900833	6902218	+		4	319	R2R3	cell wall, lignin, seed oil, axillary meristem
Ma03_g09840		7294462	7296093	+		3	320	R2R3	anther-, tapetum development
Ma03_g11910		9246422	9247790	+		4	360	R2R3	
Ma03_g12480		9621017	9623699	-		4	175	R2R3	defense, stress response
Ma03_g12720		9782781	9783893	-		4	290	R2R3	stress response, hormone signaling
Ma03_g14020		11197863	11198880	-		2	313	R2R3	suberin
Ma03_g18410		23945840	23948315	+		3	168	R2R3	cell wall, lignin, seed oil, axillary meristem
Ma03_g19810		25073787	25074642	+		2	259	R2R3	cell wall, lignin, seed oil, axillary meristem
Ma03_g20390		25556498	25558664	-		3	382	R2R3	embryogenesis, seed maturation
Ma03_g21920		26789598	26790783	+	+	5	254	R2R3	flavonoid repressor
Ma03_g21970		26827699	26828817	+		3	303	R2R3	
Ma03_g23170		27799667	27800542	-	+	4	217	R2R3	repressors PP, sinapate, lignin
Ma03_g25780		29742629	29743879	+		4	344	R2R3	mucillage, lignin, stomatal closure
Ma03_g28720		31825604	31827664	+	+	3	274	R2R3	proanthocyanidins
Ma03_g29070		32109761	32110872	-		3	325	R2R3	
Ma03_g29510		32402508	32403434	-		2	288	R2R3	stress response, hormone signaling
Ma03_g29770		32613605	32614662	+		3	307	R2R3	
Ma04_g00460		405252	406293	+		4	265	R2R3	defense, stress response
Ma04_g01010		896369	897222	-		3	226	R2R3	
Ma04_g05460		4086523	4089539	+		4	254	R2R3	photomorphogenesis
Ma04_g06410		4737396	4738484	+		3	306	R2R3	
Ma04_g09430		6706438	6707529	+		3	302	R2R3	stress response, hormone signaling
Ma04_g11930		8517172	8518916	+		3	228	R2R3	photomorphogenesis
Ma04_g12940		9788899	9789804	+		2	266	R2R3	stress response, hormone signaling
Ma04_g13260		10044518	10045599	+		3	308	R2R3	
Ma04_g16770		16592910	16594109	-		3	348	R2R3	suberin
Ma04_g18740		20887894	20888897	+		4	146	R2R3	ASR, flower morphogenesis, stilbene
Ma04_g19500		22140585	22141813	+		2	347	R2R3	suberin
Ma04_g20120		22833201	22834619	+		4	322	R2R3	anther development, stress response
Ma04_g22200		24575839	24576649	-		3	195	R2R3	repressors PP, sinapate, lignin
Ma04_g22930		25120275	25121367	-		4	254	R2R3	axillary meristem, root growth
Ma04_g23220		25400512	25401385	-		3	243	R2R3	PP, lignin
Ma04_g24670		26639014	26639915	+		3	243	R2R3	
Ma04_g26220		27753189	27754723	+		4	283	R2R3	
Ma04_g26550	MYB85 [22]	27973322	27974181	-		2	249	R2R3	secondary cell wall, lignin
Ma04_g26660		28040859	28041969	-		4	128	R2R3	defense, stress response
Ma04_g26810		28140628	28141740	-		4	269	R2R3	stress response, hormone signaling
Ma04_g28300		29374644	29376644	-	+	3	232	R2R3	
Ma04_g28510		29563855	29564811	+		4	219	R2R3	ASR, flower morphogenesis, stilbene
Ma04_g30160		30890260	30891484	-		2	368	R2R3	anther development, stress response
Ma04_g31800		32024735	32025857	+		3	279	R2R3	axillary meristem, root growth
Ma04_g31880		32081718	32084107	-		4	142	R2R3	photomorphogenesis
Ma04_g32240		32306362	32307003	+		2	187	R2R3	repressors PP, sinapate, lignin
Ma04_g33920	MYB72 [22]	33328252	33329340	+		3	300	R2R3	PP, lignin
Ma04_g34300		33575039	33576369	+		3	382	R2R3	anther development, stress response
Ma04_g34660		33734735	33735587	+		3	226	R2R3	axillary meristem, root growth
Ma04_g35350		34164151	34165124	-		3	272	R2R3	PP, lignin
Ma04_g35730		34361423	34362164	+		3	204	R2R3	PP, lignin
Ma04_g35890		34456334	34457640	-		3	279	R2R3	photomorphogenesis
Ma04_g38740		36139124	36140139	+		3	279	R2R3	axillary meristem, root growth
Ma05_g01100		651244	652022	+		3	206	R2R3	repressors PP, sinapate, lignin
Ma05_g01880		1153222	1154262	-		3	281	R2R3	axillary meristem, root growth
Ma05_g03340		2404480	2405576	+		3	303	R2R3	PP, lignin
Ma05_g03690		2718554	2719768	-		4	240	R2R3	ASR, flower morphogenesis, stilbene
Ma05_g05670		4316981	4317987	-		4	246	R2R3	secondary cell wall, lignin
Ma05_g06310		4705834	4707231	-	+	3	375	R2R3	general flavonoid, trichome
Ma05_g07140		5206984	5207790	-		1	269	R2R3	stress tolerance
Ma05_g07450		5427233	5429855	-		5	295	R2R3	defense, stress response
Ma05_g08960		6598702	6600983	+		5	192	R2R3	
Ma05_g10430		7526380	7527556	+		3	279	R2R3	axillary meristem, root growth
Ma05_g12030		8749432	8751067	+		5	255	R2R3	flower meristem identity
Ma05_g14510		10595192	10596181	-		4	229	R2R3	ASR, flower morphogenesis, stilbene
Ma05_g17720		21202263	21203407	-		3	234	R2R3	
Ma05_g18420		23864552	23865432	+		3	241	R2R3	repressors PP, sinapate, lignin
Ma05_g18710		24606320	24608917	-		5	288	R2R3	defense, stress response
Ma05_g19630		28142012	28143079	-		3	295	R2R3	root development
Ma05_g20320		31975647	31976748	+		3	307	R2R3	suberin
Ma05_g20740		32405906	32411144	+		6	375	R2R3	
Ma05_g20940		32642222	32646244	+		3	268	R2R3	
Ma05_g23480		35547417	35548386	+		4	182	R2R3	defense, stress response
Ma05_g23640		35794926	35797163	-		3	321	R2R3	flavonols, phlobaphene
Ma05_g24200		36482627	36483576	+		3	207	R2R3	axillary meristem, root growth
Ma05_g24840		36981509	36982626	+		3	323	R2R3	anther-, tapetum development
Ma05_g25150		37166050	37167522	+		3	316	R2R3	axillary meristem, root growth
Ma05_g25490		37423048	37423913	-		3	226	R2R3	secondary cell wall, lignin
Ma05_g25630		37499891	37501199	-		6	290	R2R3	trichome branching, petal morphogenesis
Ma05_g25680		37532411	37533682	+		3	302	R2R3	cell wall, lignin, seed oil, axillary meristem
Ma05_g28370		39396363	39402499	-	+	5	210	R2R3	repressors PP, sinapate, lignin
Ma05_g30120		40637530	40638485	+		3	252	R2R3	cell wall, lignin, seed oil, axillary meristem
Ma05_g30720		40993457	40994353	+		3	237	R2R3	cell cycle regulation
Ma05_g31160		41195712	41202456	-		4	397	R2R3	stress tolerance
Ma05_g31440		41356727	41357758	+		1	344	R2R3	leaf-, shoot-, germ morphogenesis
Ma06_g00910		744618	747868	+		5	394	R2R3	cell wall, lignin, seed oil, axillary meristem
Ma06_g03570		2604334	2605906	+		3	333	R2R3	axillary meristem, root growth
Ma06_g04210		3055555	3059353	-	+	4	361	R2R3	proanthocyanidins
Ma06_g04240		3072400	3074637	-		7	345	R2R3	trichome branching, petal morphogenesis
Ma06_g04270		3095165	3096766	+		4	354	R2R3	cell wall, lignin, seed oil, axillary meristem
Ma06_g04370		3152489	3153586	+		4	250	R2R3	defense, stress response
Ma06_g05680		4227458	4228553	+		3	305	R2R3	root development
Ma06_g05960		4396784	4397869	-	+	3	275	R2R3	anthocyanins
Ma06_g06660		4801530	4808116	-		7	517	3R	cell cycle control
Ma06_g08100		5748553	5749986	+		3	276	R2R3	embryogenesis, seed maturation
Ma06_g08440		5976699	5977672	-		3	243	R2R3	
Ma06_g08910		6239164	6240262	+	+	5	199	R2R3	repressors PP, sinapate, lignin
Ma06_g11140	MaMYB3 [23]	7827980	7828834	+	+	4	200	R2R3	starch degradation, flavonoid repressor
Ma06_g11270		7905384	7906681	+		3	308	R2R3	defense, stress response
Ma06_g12110		8408418	8411546	-		12	469	R2R3	guard cell division, root gravitropism
Ma06_g12160		8449881	8450871	-		4	256	R2R3	defense, stress response
Ma06_g14470		9914817	9917018	-	+	5	286	R2R3	repressors PP, sinapate, lignin
Ma06_g16350		11053657	11054699	+	+	3	290	R2R3	general flavonoid, trichome
Ma06_g16920		11470816	11471624	+		3	220	R2R3	cell cycle regulation
Ma06_g17440		11851435	11852673	-		4	275	R2R3	cell wall, lignin, seed oil, axillary meristem
Ma06_g19030		13020150	13021197	+		2	263	R2R3	cell wall, lignin, seed oil, axillary meristem
Ma06_g27210		29243310	29244238	-		4	237	R2R3	repressors PP, sinapate, lignin
Ma06_g29060		30553280	30554423	+		3	195	R2R3	stamen development
Ma06_g31020		32237208	32238426	+		3	351	R2R3	defense, stress response
Ma06_g32530		33447718	33449121	+		3	315	R2R3	axillary meristem, root growth
Ma06_g33100		33858326	33871293	+		12	838	4R	SNAP complex
Ma06_g33190		33914360	33915233	+		3	244	R2R3	
Ma06_g33430		34073015	34074005	+		3	273	R2R3	axillary meristem, root growth
Ma06_g33920		34371289	34372730	+		3	346	R2R3	cell wall, lignin, seed oil, axillary meristem
Ma06_g35430		35255099	35260321	+		3	225	R2R3	stress tolerance
Ma06_g35620		35399156	35400628	+		4	400	R2R3	embryogenesis, seed maturation
Ma06_g37660		36662931	36664093	+		3	299	R2R3	anther-, trichome development
Ma06_g38880		37507220	37508388	-		3	311	R2R3	anther-, trichome development
Ma07_g00270		249167	250371	+		2	361	R2R3	
Ma07_g02470		1968784	1973645	-		3	600	R2R3	anther development, stress response
Ma07_g05660		4114936	4116029	+		3	272	R2R3	defense, stress response
Ma07_g05780		4200621	4201699	+		2	251	R2R3	secondary cell wall, lignin
Ma07_g08110		6059949	6061103	+		5	256	R2R3	axillary meristem, root growth
Ma07_g10330		7698721	7708970	+		7	568	3R	cell cycle control
Ma07_g10340		7710266	7711261	-		1	332	R2R3	leaf-, shoot-, germ morphogenesis
Ma07_g11110		8262980	8264078	-		3	187	R2R3	cell wall, lignin, seed oil, axillary meristem
Ma07_g12330		9212653	9213850	+		4	272	R2R3	secondary cell wall, lignin
Ma07_g13590		10213901	10216977	-	+	3	307	R2R3	general flavonoid, trichome
Ma07_g17600		20759044	20760488	+		5	343	R2R3	
Ma07_g19350		27376443	27377703	-		5	270	R2R3	defense, stress response
Ma07_g19470		27474578	27475521	+		4	241	R2R3	cell cycle regulation
Ma07_g19700		27655399	27656957	-		2	470	R2R3	cell wall, lignin, seed oil, axillary meristem
Ma07_g19720		27682692	27684725	+		6	357	R2R3	trichome branching, petal morphogenesis
Ma07_g19880 *		27793556	27794919	+	+	4	264	R2R3	proanthocyanidins
Ma07_g19890 *		27798326	27799369	-	+	3	269	R2R3	proanthocyanidins
Ma07_g20020		27925622	27926695	+		4	259	R2R3	secondary cell wall, lignin
Ma07_g20990		28973984	28975550	-		3	341	R2R3	axillary meristem, root growth
Ma07_g22540		30450574	30451644	-		4	269	R2R3	defense, stress response
Ma07_g23060		30809797	30823608	+		3	551	R2R3	anther development, stress response
Ma07_g23180		30932263	30933303	+		3	289	R2R3	defense, stress response
Ma07_g23230 *		30951709	30952887	+		3	274	R2R3	cell wall, lignin, seed oil, axillary meristem
Ma07_g23240 *		30951829	30952875	+		3	230	R2R3	cell wall, lignin, seed oil, axillary meristem
Ma07_g26530		33294011	33295448	-		4	292	R2R3	
Ma08_g01300		1210795	1211973	-		5	263	R2R3	defense, stress response
Ma08_g01760		1466390	1467139	+		1	250	R2R3	stress tolerance
Ma08_g02100		1707379	1708990	+		3	421	R2R3	embryogenesis, seed maturation
Ma08_g02450		1904860	1905752	+		3	241	R2R3	
Ma08_g03420		2489605	2490759	+		3	319	R2R3	anther-, trichome development
Ma08_g10260		7475942	7478109	-		3	371	R2R3	flavonols, phlobaphene
Ma08_g10600		7758649	7759595	-		3	256	R2R3	defense, stress response
Ma08_g11120		8204512	8205519	+		3	278	R2R3	photomorphogenesis
Ma08_g12510		9469693	9470558	+		3	231	R2R3	PP, lignin
Ma08_g13070		10389299	10390584	-		3	313	R2R3	axillary meristem, root growth
Ma08_g14720		14652484	14653886	+		4	370	R2R3	
Ma08_g15820		16034645	16037684	-	+	3	260	R2R3	
Ma08_g15960		16626283	16635800	-		3	557	R2R3	
Ma08_g16760		20724484	20725190	-	+	3	194	R2R3	flavonoid repressor
Ma08_g17860		27301472	27302427	-		3	249	R2R3	defense, stress response
Ma08_g18540		32070622	32071809	-		4	276	R2R3	defense, stress response
Ma08_g23390		36792861	36793894	+		3	290	R2R3	proanthocyanidins
Ma08_g25570		38350630	38352260	+		3	307	R2R3	axillary meristem, root growth
Ma08_g25960	MYBS3 [24]	38629624	38630727	+		4	296	R2R3	stress response, hormone signaling
Ma08_g26720		39203311	39211401	-		6	541	R2R3	
Ma08_g30360		41652620	41653601	+		3	269	R2R3	defense, stress response
Ma08_g31720	MYB83 [22]	42548860	42549849	+		3	279	R2R3	secondary wall, lignin
Ma08_g32760		43364702	43366041	+		4	200	R2R3	repressors PP, sinapate, lignin
Ma08_g34230		44310614	44311366	-	+	3	203	R2R3	repressors PP, sinapate, lignin
Ma08_g34710		44706501	44707468	+		3	245	R2R3	
Ma09_g03310		2240622	2241647	-		1	342	R2R3	leaf-, shoot-, germ morphogenesis
Ma09_g03740		2481273	2483877	+		2	322	R2R3	stress tolerance
Ma09_g04930		3159224	3160371	-		3	302	R2R3	cell wall, lignin, seed oil, axillary meristem
Ma09_g06730		4303577	4304525	+		3	204	R2R3	stamen development
Ma09_g08140		5353783	5354623	+		2	256	R2R3	cell wall, lignin, seed oil, axillary meristem
Ma09_g08260		5451934	5452919	-	+	3	272	R2R3	repressors PP, sinapate, lignin
Ma09_g09400		6192942	6193897	-		3	263	R2R3	
Ma09_g09720		6398143	6410415	-		13	798	4R	SNAP complex
Ma09_g10800		7342974	7344121	-		3	327	R2R3	anther-, trichome development
Ma09_g11770		8000924	8002091	-		4	262	R2R3	defense, stress response
Ma09_g13170		8908685	8910010	-		3	310	R2R3	axillary meristem, root growth
Ma09_g14260		9743683	9745180	-		4	187	R2R3	root development
Ma09_g15050		10356705	10357805	-		4	248	R2R3	secondary cell wall, lignin
Ma09_g15130		10456847	10458264	+		4	211	R2R3	cell wall, lignin, seed oil, axillary meristem
Ma09_g15440		10764272	10765275	-		4	257	R2R3	proanthocyanidins
Ma09_g15940		11297314	11298296	-		3	260	R2R3	defense, stress response
Ma09_g16940		12438026	12439426	+		4	279	R2R3	defense, stress response
Ma09_g16980		12476657	12478055	+		4	304	R2R3	cell wall, lignin, seed oil, axillary meristem
Ma09_g20280		28998021	28999222	+		3	273	R2R3	axillary meristem, root growth
Ma09_g22730		34631677	34632781	-		3	315	R2R3	
Ma09_g23100		35024484	35029665	+		4	288	R2R3	stress response, hormone signaling
Ma09_g23770		35511400	35518711	+		5	1120	2R	CDC5
Ma09_g24640	MYB31 [21]	36291996	36292655	-		3	173	R2R3	repressors PP, sinapate, lignin
Ma09_g25010		36615326	36616400	+		3	301	R2R3	defense, stress response
Ma09_g25590		36999501	37000692	-		3	323	R2R3	anther-, trichome development
Ma09_g27990		38852584	38853631	-	+	3	260	R2R3	anthocyanins
Ma09_g28970		39592459	39593487	+		1	343	R2R3	leaf-, shoot-, germ morphogenesis
Ma09_g29010		39617399	39618193	-		3	214	R2R3	
Ma09_g29660	MYB52 [22]	40061204	40062015	-		3	210	R2R3	cell wall, lignin, seed oil, axillary meristem
Ma10_g01730		5102446	5104125	-		5	286	R2R3	defense, stress response
Ma10_g01750		5159022	5160613	+		5	288	R2R3	defense, stress response
Ma10_g04420		15095718	15097543	-		5	284	R2R3	
Ma10_g04920		15562156	15563431	-		3	321	R2R3	cell wall, lignin, seed oil, axillary meristem
Ma10_g05260		15989100	15989828	+		1	243	R2R3	stress tolerance
Ma10_g05680		17016063	17017842	+		3	499	R2R3	embryogenesis, seed maturation
Ma10_g06140		17599985	17600773	+		3	212	R2R3	
Ma10_g09100		23301527	23306607	-		4	297	R2R3	anther-, trichome development
Ma10_g09370		23565764	23566747	-		3	268	R2R3	axillary meristem, root growth
Ma10_g10820		24568790	24569501	+		3	176	R2R3	repressors PP, sinapate, lignin
Ma10_g11100		24705755	24706780	-		3	260	R2R3	cell wall, lignin, seed oil, axillary meristem
Ma10_g13000		25942170	25943424	+		4	304	R2R3	cell wall, lignin, seed oil, axillary meristem
Ma10_g13640		26378393	26379280	-		3	236	R2R3	cell cycle regulation
Ma10_g14150		26690247	26691396	-	+	4	298	R2R3	general flavonoid, trichome
Ma10_g14950		27211124	27223165	+		16	869	3R	cell cycle control
Ma10_g16050		27917472	27918339	+	+	3	217	R2R3	repressors PP, sinapate, lignin
Ma10_g17650		28964249	28965405	+	+	3	278	R2R3	anthocyanins
Ma10_g18840		29621056	29624189	+		12	467	R2R3	guard cell division, root gravitropism
Ma10_g19130		29806495	29807615	-		3	297	R2R3	root development
Ma10_g19820		30234061	30235019	-		4	175	R2R3	defense, stress response
Ma10_g19970		30309772	30312309	-	+	4	206	R2R3	flavonoid repressor
Ma10_g24510		33076512	33077586	-		4	305	R2R3	root development
Ma10_g25660		33694709	33695874	+		3	314	R2R3	root development
Ma10_g26540		34187707	34195688	+		7	567	3R	cell cycle control
Ma10_g26660		34247924	34248785	+		3	226	R2R3	
Ma10_g29230		35877509	35878859	-		5	315	R2R3	defense, stress response
Ma10_g29290		35933319	35936352	-		3	562	R2R3	anther development, stress response
Ma10_g29660		36170205	36171708	-		7	289	R2R3	trichome branching, petal morphogenesis
Ma10_g29900		36334814	36335832	+		3	285	R2R3	secondary cell wall, lignin
Ma11_g00330		235287	236166	-		3	235	R2R3	
Ma11_g00350		255492	257081	+		6	375	R2R3	
Ma11_g02310		1659794	1660627	-		3	213	R2R3	PP, lignin
Ma11_g03860		2954888	2956813	-		4	294	R2R3	flower meristem identity
Ma11_g04680		3650672	3651636	+		3	264	R2R3	defense, stress response
Ma11_g06880		5505145	5505826	-		3	167	R2R3	repressors PP, sinapate, lignin
Ma11_g07330		5826847	5827930	+		3	261	R2R3	axillary meristem, root growth
Ma11_g07530		6014287	6015836	-		3	357	R2R3	suberin
Ma11_g08730		6941094	6942543	-		4	275	R2R3	cell wall, lignin, seed oil, axillary meristem
Ma11_g10680		10271192	10273848	-		6	275	R2R3	defense, stress response
Ma11_g10710		10304514	10305532	+		3	275	R2R3	
Ma11_g11300		12744937	12748426	+		6	251	R2R3	defense, stress response
Ma11_g11940		15469544	15470676	-		3	309	R2R3	defense, stress response
Ma11_g14670		20385602	20386542	-		3	262	R2R3	defense, stress response
Ma11_g15740		21412108	21413062	-		4	230	R2R3	PP, lignin
Ma11_g16150		21711896	21713795	-		5	233	R2R3	
Ma11_g16430		21944276	21945343	+		3	287	R2R3	PP, lignin
Ma11_g19220		24164576	24166138	-		4	344	R2R3	defense, stress response
Ma11_g21160		25411345	25412681	-		6	303	R2R3	
Ma11_g21730		25754364	25755420	-		2	326	R2R3	suberin
Ma11_g21820		25814103	25816846	-	+	3	235	R2R3	
Ma11_g23010		26544741	26545608	-		3	241	R2R3	cell cycle regulation
Ma11_g23420		26767696	26769125	+		3	183	R2R3	cell wall, lignin, seed oil, axillary meristem
Ma00_g01590		9744573	9745661	+		2	283	R2R3	stress response, hormone signaling
Ma00_g04340		36355056	36356428	+		2	283	R2R3	secondary wall, lignin
Ma00_g04960		43098834	43099818	-		3	267	R2R3	defense, stress response

The genes are ordered by DH Pahang version 2 [18] pseudochromosomes, from north to south. The annotation-version specific gene code describing the chromosomal assignment and position on pseudochromosomes is given in the first column. "Ma00" indicates genes located in sequences without chromosomal assignment. Functional assignment is based on the Maximum Likelihood tree presented in Fig 2. Multi-FASTA files with *MaMYB* CDS and peptide sequences together with a general feature format (GFF) file describing the annotated *MaMYB* genes is provided in the supplementaries. str.: strand

*: paralogs, ASR: abiotic stress response, PP: phenylpropanoid.

The number of R2R3-MYB genes is one of the highest among the species that have been studied to date, ranging from 45 in *Ginkgo biloba* [10] over 157 in *Zea mays* [25] and 249 in *Brassica napus* [26] to 360 in *Gossypium hirsutum* [11]. This is probably due to three whole-genome duplications (γ 100 Myr ago and α, β 65 Myr ago) that occurred during *Musa* genome evolution [3, 27]. The number of atypical multiple repeat *MYB* genes identified in *M*. *acuminata* is in the same range as those reported for most other plant species, up to six *MYB3R* and up to two *MYB4R* and *CDC5*-like genes.

The 285 *MaR2R3-MYB* genes identified constitute approximately 0.81% of the 35,276 predicted protein-coding *M*. *acuminata* genes and 9.0% of the 3,155 putative *M*. *acuminata* transcription factor genes [18]. These were subjected to further analyses. The identified *MaR2R3-MYB* genes were named following the nomenclature of the locus tags provided in the DH-Pahang version 2 genome annotation (Table 1). A keyword search in the NCBI database (http://www.ncbi.nlm.nih.gov/) revealed no evidence for *M*. *acuminata MYB* genes not present in Table 1.

Six publications dealing with *M*. *acuminata MYB* genes were identified: one study describes the elevated expression of nine *MaMYB* genes in transgenic banana plants overexpressing the NAC domain transcription factor MusaVND1 (vascular related NAC domain) indicating a role of these MaMYBs in the regulation of secondary wall deposition [22]. MYBS3 *(Ma08_25960)* was found to be differentially expressed between cold-sensitive and cold-tolerant bananas [24]. Another publication described the *M*. *acuminata R2R3-MYB* gene *Ma05_03690* being upregulated in the early response to the endoparasitic root-knot nematode *Meloidogyne incognita* in roots [28]. MusaMYB31 *(Ma01_02850)* was identified as a negative regulator of lignin biosynthesis and the general phenylpropanoid biosynthesis pathway [21]. MaMYB3 *(Ma06_11140)* was found to repress starch degradation in fruit ripening [23] and MaMYB4 *(Ma01_19610)* was recently described to control fatty acid desaturation [29].

**Fig 1:**
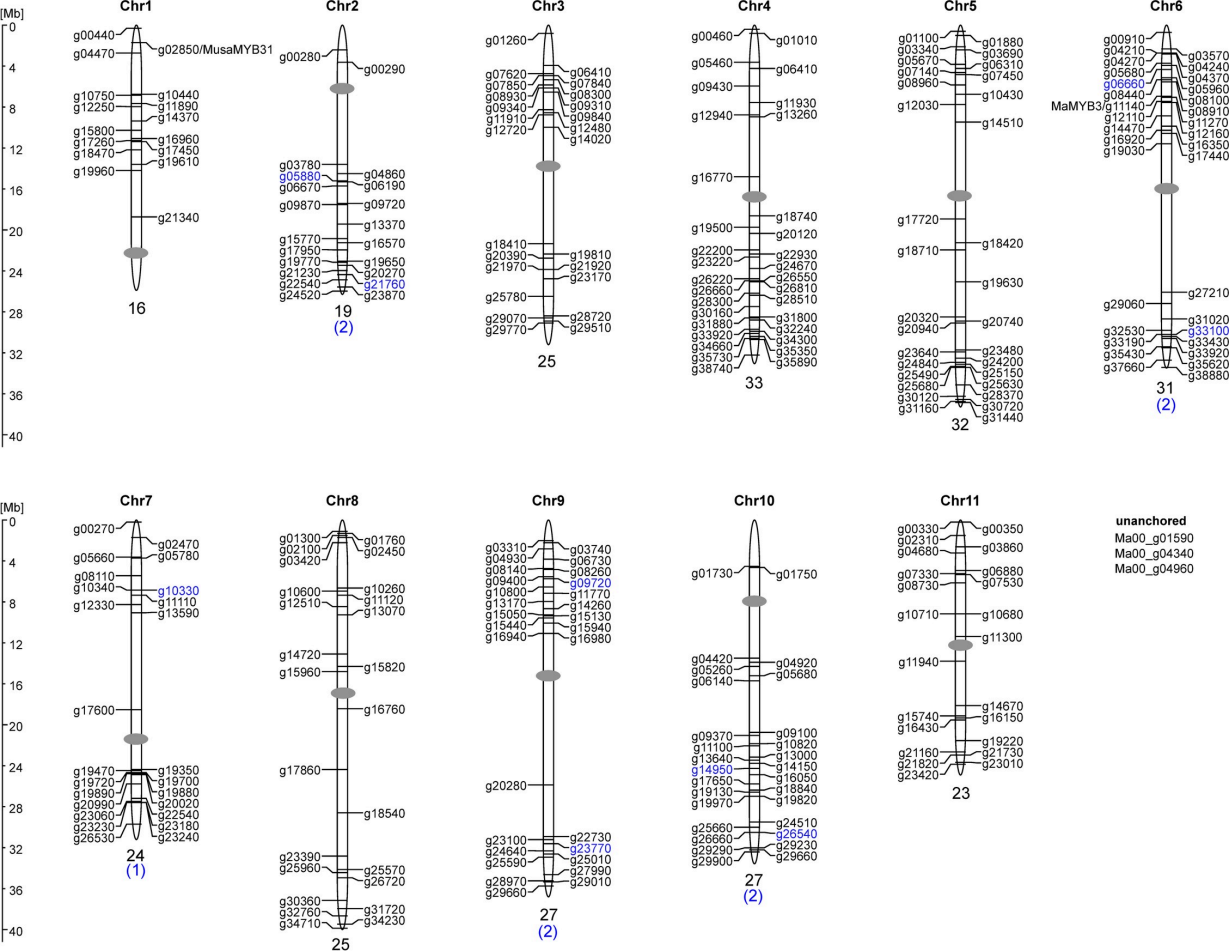
Distribution of *MaMYB* genes on the eleven *M*. *acuminata* chromosomes.

On the basis of the DH-Pahang version 2 annotation, 291 of the 294 *MaMYB* genes could be assigned to the eleven chromosomes. The chromosomal distribution of *MaMYB* genes on the pseudochromosomes is shown in Fig 1 and revealed that *M*. *acuminata MYB* genes are distributed across all chromosomes.

Gene structure analysis revealed that most *MaR2R3-MYB* genes (155 of 285; 54.4%) follow the previously reported rule of having two introns and three exons, and display the highly conserved splicing arrangement that has also been reported for other plant species [25, 30, 31]. A total of 23 (8.1%) *MaR2R3-MYB* genes have two exons and seven (2.5%) were single exon genes. 67 (23.5%) *MaR2R3-MYB* genes have four exons, 21 (7.4%) five exons, eight (2.8%) six exons and two of each with seven and twelve exons, respectively. The complex exon-intron structure of Ma06_12110 and Ma10_18840 is conserved in their *A*. *thaliana* orthologs AtMYB88 and AtMYB124/FOUR LIPS (FLP) containing ten and eleven exons, respectively. This supports their close evolutionary relationship, but also suggests the conservation of this intron pattern in evolution since the monocot-dicot split 140–150 Myr ago [32].

Chromosomes are drawn to scale. The positions of centromeres (grey ovals) are roughly estimated from repeat distribution data. The chromosomal positions of the *MaMYB* genes (given in DH Pahang version 2 annotation ID) are indicated. *R2R3-MYB* genes are given in black letters, *MYB* genes with more than two MYB repeats are given in blue letters. The number of *MYB* genes on each chromosome is given below the respective chromosome.

### Phylogenetic analysis of the *M*. *acuminata* MYB family

With the aim to explore the putative function of the predicted *M*. *acuminata* MYBs, we assigned them to plant MYB proteins with known function. For this, we chose primarily data from *A*. *thaliana*, which is the source of most functional MYB characterisations. From comparable studies, MYB function appears conserved across MYB clades, suggesting that closely related MYBs recognise similar/same target genes and possess cooperative, overlapping or redundant functions.

**Fig 2:**
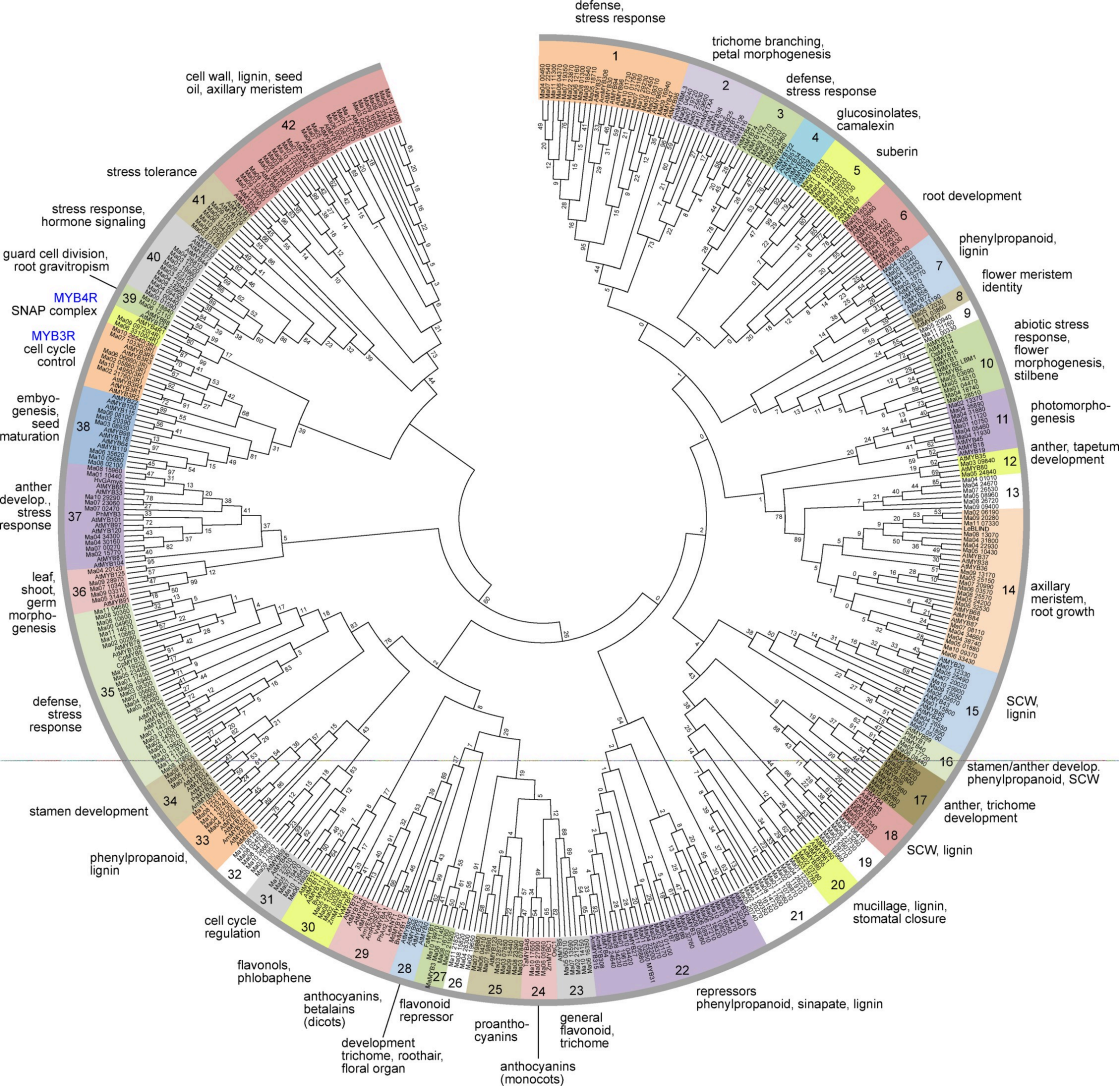
Phylogenetic Maximum Likelihood (ML) tree.

To unravel the relationships, we constructed a phylogenetic tree with 468 MYB domain amino acid sequences of MYB proteins. We used 293 MaMYBs (omitting the CDC5-like MaMYB), the complete *A*. *thaliana* MYB family (132 members, including 126 R2R3-MYB, five MYB3R and one MYB4R) and 43 functionally well characterised landmark R2R3-MYBs from other plant species. The phylogenetic tree topology allowed us to classify the analysed MYBs into one MYB3R clade, one MYB4R clade and 42 R2R3-MYB protein clades (Fig 2).

Most R2R3-MYB clades (31 of 42) include variable numbers of MYB proteins from Arabidopsis and banana, indicating that the appearance of most *MYB* genes in these two species predates the monocot-dicot split as observed in other studies [33]. Several of these clades also contain landmark MYBs from other plant species. The two clades 4 and 28 only contain Arabidopsis R2R3-MYB members, while seven clades (9, 13, 19, 21, 26, 29 and 32, displayed with white background in Fig 2) only contain banana R2R3-MYB members. Additionally, two clades, 24 with monocot anthocyanin biosynthesis regulators and 27 with the strawberry landmark flavonoid biosynthesis repressor FaMYB1 [34], do not contain any *A*. *thaliana* MYB (Fig 2).

ML consensus tree inferred from 1000 bootstraps with 468 MYB domain amino acid sequences of MYB proteins from *Musa accuminata* (Ma), *Arabidopsis thaliana* (At) and landmark MYBs from other plant species built with MEGA7. Clades are labeled with different colors and functional annotations are given. The numbers at the branches give bootstrap support values from 1000 replications. SCW, secondary cell wall.

The lineage specificity of some MYB clades could indicate that these clades may have been lost or gained in a single order or species during plant evolution, as indicated by other studies [35–37]. For example, clade 28 lacked *M*. *acuminata* orthologs, but includes the *A*. *thaliana* R2R3-MYBs AtMYB0/GLABRA1 and AtMYB66/WEREWOLF, which have been identified as being involved in the formation of trichomes and root hairs from epidermal cells [38, 39]. Similar observations have been made in maize (monocot) and sugarbeet (eudicot, caryophyllales) [30, 31], both not containing clade 28 orthologs, while grape (eudicot, rosid) and poplar (eudicot, rosid) do [12, 40]. It has been hypothesized that GLABRA1-like *MYB* genes have been acquired in rosids after the rosid-asterids split [41]. The absence of MaMYBs in clade 28 is consistent with this hypothesis, since monocots branched off before the separation of asterids and rosids in eudicots. Clade 4 also lacks banana R2R3-MYBs. This clade contains the glucosinolate biosynthesis regulators AtMYB28, AtMYB34 and AtMYB51 [42, 43]. The absence of MaMYBs in this clade is concordant with the fact that glucosinolates are only present in the Brassicaceae family. This clade is thought to have originated from a duplication event before the divergence of the genus Arabidopsis from Brassica [44].

The seven clades containing only MaMYBs were manually inspected by applying BLAST searches at the NCBI protein database in order to identify high similarity to functionally characterized landmark plant R2R3-MYBs. In no case could a landmark MYB be identified. Consequently, these clades could be described as a lineage-specific expansion in *M*. *acuminata*, reflecting a species-, genus- or order-specific evolutionary change. These MaMYB proteins may have specialised functions that were acquired or expanded in *M*. *acuminata* during genome evolution. Further research will be needed to decipher the biological roles of these *MaMYB* genes.

R2R3-MYBs may interact with basic helix-turn-helix (bHLH)-type transcription factors, together with WD-repeat (WDR) proteins, forming a trimeric MBW complex. These R2R3-MYBs are defined by a bHLH-binding consensus motif [D/E]Lx2[R/K]x3Lx6Lx3R [45] found in all bHLH-interacting R2R3-MYBs. A search in the MaMYB proteins for the mentioned bHLH-interaction motif identified 30 MaMYBs containing this motif (Table 1) and thus putatively interacting with bHLH proteins. 26 of these 30 MaMYBs were all functionally assigned to clades containing (potentially) known bHLH-interacting R2R3-MYBs: nine in clade 22 (repressors phenylpropanoid, sinapate-, lignin regulators), six in clade 25 (proanthocyanidin regulators), four in clade 23 (general flavonoid, trichome regulators), four in clade 27 (flavonoid repressors) and three in clade 24 (monocot anthocyanin regulators). Four MaMYBs, Ma02_19650, Ma04_28300, Ma08_15820 and Ma11_21820 were all found in the *M*. *acuminata*-specific clade 26. These MaMYBs may interact with bHLH-type transcription factors. The close relation to flavonoid biosynthesis regulators suggests that they may act similarly to R2R3-MYB proanthocyanidin regulators. Further research is needed to determine if they regulate a *Musa*-specific biosynthesis pathway.

### Expression profiles for *M*. *acuminata MYB* genes in different organs and developmental stages

Since it is not unusual for large transcription factor families in higher organisms to have redundant functions, a particular transcription factor needs to be studied and characterised in the context of the whole family. In this regard, the gene expression pattern can provide a hint to gene function. In this study we used publicly-available RNA-Seq reads from the Sequence Read Archive (S2 Table) to analyse the expression of the 293 *MaMYB* genes in different organs and developmental stages: embryonic cell suspension, seedling, root and young, adult and old leaf, pulp stages S1-S4 and peel stages S1-S4. Filtered RNA-Seq reads were aligned to the reference genome sequence and the number of mapped reads per annotated transcript were quantified and compared across the analysed samples to calculate normalised RNA-Seq read values which are given in Table 2.

**Table 2:** The expression profiles of *MaR2R3-MYB* genes in different organs and developmental stages based on RNA-Seq data.

gene	embryogenic	seedling	root	leaf	young_leaf	adult_leaf	old_leaf	pulp	pulpS1	pulpS2	pulpS3	pulpS4	peel	peelS1	peelS2	peelS3	peelS4
Ma00_g01590	50	35	6	151	77	175	348	6	5	5	8	5	8	2	19	10	2
Ma00_g04340	0	0	0	0	0	0	0	0	1	0	0	0	0	0	0	0	0
Ma00_g04960	0	0	0	0	0	0	0	0	0	0	0	0	0	0	0	0	0
Ma01_g00440	181	49	38	129	53	175	276	32	31	58	34	7	30	9	63	35	11
Ma01_g02850	9	2	14	27	21	31	38	20	24	23	23	10	11	7	9	17	12
Ma01_g04470	1	0	12	4	1	6	2	9	13	9	7	8	6	6	3	7	7
Ma01_g10440	3	16	4	5	7	5	5	4	5	5	3	3	3	3	2	3	4
Ma01_g10750	0	0	2	2	0	0	4	1	1	1	1	2	4	5	4	4	5
Ma01_g11890	0	0	3	3	10	1	0	0	0	0	0	0	0	0	0	0	0
Ma01_g12250	1	0	4	5	17	2	0	3	4	3	2	2	4	4	4	3	3
Ma01_g14370	0	17	11	0	0	0	0	5	7	7	8	0	8	3	7	18	4
Ma01_g15800	1	0	4	1	0	0	0	4	2	5	7	3	6	8	5	2	9
Ma01_g16960	0	0	1	0	1	0	0	0	0	0	0	0	0	0	0	0	0
Ma01_g17260	1	0	11	1	0	0	0	3	3	3	2	3	4	5	3	4	5
Ma01_g17450	0	0	6	2	0	1	6	5	13	2	2	1	1	1	2	2	0
Ma01_g18470	0	0	0	0	0	0	0	0	1	0	0	0	1	2	1	0	0
Ma01_g19610	13	0	6	39	56	40	57	29	78	22	15	2	12	7	36	2	5
Ma01_g19960	35	1	6	97	94	148	145	5	7	6	5	4	19	5	58	10	4
Ma01_g21340	0	0	0	0	0	0	0	2	8	0	0	0	0	0	0	0	0
Ma02_g00280	1	1	21	4	1	1	5	6	8	10	4	2	5	5	4	5	7
Ma02_g00290	0	0	3	9	5	26	1	8	25	0	6	2	3	2	3	2	5
Ma02_g03780	0	0	29	18	26	0	0	13	14	9	18	12	30	39	31	17	33
Ma02_g04860	0	0	1	0	0	0	0	0	0	1	0	0	0	0	0	0	0
Ma02_g05880	4	2	2	3	2	5	3	2	4	2	2	1	2	1	1	1	3
Ma02_g06190	0	0	19	8	0	0	0	16	30	12	13	11	28	42	18	18	32
Ma02_g06670	1	1	4	1	0	0	0	4	3	7	4	4	5	6	2	6	5
Ma02_g09720	0	0	0	0	0	0	0	0	0	0	0	0	0	1	0	0	1
Ma02_g09870	29	1	23	37	99	33	11	19	15	28	27	6	11	11	10	9	13
Ma02_g13370	0	0	1	0	0	0	0	0	0	0	0	0	0	0	0	0	0
Ma02_g15770	6	0	1	0	0	1	0	1	2	0	1	0	1	1	1	0	1
Ma02_g16570	0	0	0	0	1	0	1	0	0	0	0	0	0	0	0	0	0
Ma02_g17950	8	0	46	14	4	6	13	33	53	35	16	26	19	19	14	20	22
Ma02_g19650	0	0	3	3	5	1	0	7	10	5	3	11	9	15	8	6	5
Ma02_g19770	0	0	0	0	0	0	0	0	0	0	0	0	0	0	0	0	0
Ma02_g20270	3	0	3	16	57	4	0	1	1	2	2	1	4	3	4	3	7
Ma02_g21230	2	2	1	5	14	3	1	3	8	3	0	0	1	0	3	2	0
Ma02_g21760	0	3	7	2	2	0	0	8	11	7	8	7	8	11	5	5	12
Ma02_g22540	1	0	7	4	2	6	7	1	1	1	2	0	1	1	1	1	1
Ma02_g23870	3	0	0	7	4	20	3	2	8	0	0	0	0	0	0	0	0
Ma02_g24520	0	0	0	0	0	0	0	0	1	0	0	0	0	0	0	0	0
Ma03_g01260	11	0	6	16	0	0	61	9	31	3	1	2	2	1	3	4	1
Ma03_g06410	0	0	11	1	0	0	0	1	0	3	0	1	1	2	0	2	1
Ma03_g07620	0	0	3	0	0	0	0	0	0	0	0	0	0	0	0	0	0
Ma03_g07840*	0	0	2	1	2	0	0	10	24	9	3	5	2	1	3	1	2
Ma03_g07850*	0	0	1	1	1	0	0	1	2	1	0	1	2	2	2	3	1
Ma03_g08300	0	1	3	0	0	0	1	1	5	0	0	0	1	0	0	6	0
Ma03_g08930	0	0	0	0	0	0	0	0	0	0	0	0	0	0	0	0	0
Ma03_g09310	0	0	0	12	42	7	1	0	0	0	0	0	0	0	0	0	0
Ma03_g09340	2	0	7	1	1	0	1	5	8	4	5	2	3	4	2	3	5
Ma03_g09840	0	0	0	0	0	0	0	0	0	0	0	0	0	0	0	0	0
Ma03_g11910	5	8	25	35	76	25	6	12	10	13	12	13	24	26	30	18	22
Ma03_g12480	0	0	4	0	0	0	0	1	1	1	0	0	0	0	0	1	0
Ma03_g12720	16	10	4	45	59	49	70	2	2	2	2	3	18	2	32	35	3
Ma03_g14020	0	0	1	0	0	0	0	2	1	4	1	3	2	3	1	3	2
Ma03_g18410	0	0	0	1	2	0	0	4	15	0	0	0	0	0	0	0	0
Ma03_g19810	0	0	2	0	0	0	0	0	0	0	0	0	0	0	0	0	0
Ma03_g20390	5	1	1	2	2	0	4	2	1	3	2	2	0	1	0	0	1
Ma03_g21920	5	0	2	17	13	32	22	4	12	2	2	0	2	0	6	3	0
Ma03_g21970	0	0	1	0	0	0	0	0	0	0	0	0	0	0	0	0	0
Ma03_g23170	0	0	3	4	8	2	0	2	2	3	1	2	4	6	2	3	4
Ma03_g25780	3	2	60	18	14	3	0	37	28	81	18	22	40	57	29	26	48
Ma03_g28720	3	3	1	2	0	1	5	1	3	1	0	1	0	0	0	0	0
Ma03_g29070	0	0	1	2	4	2	0	1	1	0	3	0	2	1	2	1	2
Ma03_g29510	14	4	5	44	49	65	55	9	25	5	5	2	6	3	7	11	3
Ma03_g29770	1	0	3	1	1	0	0	2	3	4	0	2	1	2	1	1	2
Ma04_g00460	2	0	1	9	14	20	2	0	0	0	0	0	1	0	2	1	0
Ma04_g01010	0	0	7	0	0	0	1	1	3	1	1	0	1	1	1	0	1
Ma04_g05460	0	0	0	0	0	0	0	0	0	0	1	0	1	0	1	0	1
Ma04_g06410	0	1	1	0	1	0	0	0	1	0	0	0	0	0	0	0	0
Ma04_g09430	4	1	9	12	17	19	2	4	4	2	6	5	8	11	7	3	11
Ma04_g11930	0	0	0	0	0	0	0	0	0	0	0	0	0	0	0	0	0
Ma04_g12940	4	0	2	3	6	2	4	2	3	1	2	0	1	0	1	0	0
Ma04_g13260	0	2	2	1	1	1	2	2	3	2	3	1	0	0	0	0	0
Ma04_g16770	0	1	1	1	0	1	0	1	0	1	0	3	1	1	0	1	0
Ma04_g18740	11	1	7	2	1	1	5	2	0	0	6	0	1	0	1	2	0
Ma04_g19500	0	0	4	1	0	0	0	2	4	3	0	1	1	1	1	1	0
Ma04_g20120	0	0	0	0	0	0	0	0	0	0	0	0	0	0	0	0	0
Ma04_g22200	0	0	3	0	0	0	0	3	2	8	1	3	1	1	0	1	0
Ma04_g22930	0	0	4	1	0	0	0	2	2	3	0	2	4	6	2	3	4
Ma04_g23220	0	0	5	3	0	0	1	1	3	1	1	1	4	3	6	1	6
Ma04_g24670	0	0	33	6	0	0	0	24	32	24	19	19	17	22	9	16	19
Ma04_g26220	0	1	1	2	1	1	0	1	3	1	1	0	4	2	11	2	1
Ma04_g26550	0	0	5	1	1	0	0	2	3	3	1	1	1	1	1	1	3
Ma04_g26660	0	2	22	1	0	1	3	12	27	11	5	4	1	0	2	1	2
Ma04_g26810	41	78	15	153	258	145	185	39	35	74	32	17	33	13	54	50	14
Ma04_g28300	0	0	0	0	0	0	0	0	0	0	0	0	0	0	0	0	0
Ma04_g28510	8	1	3	11	2	22	10	9	13	8	14	2	3	2	5	3	3
Ma04_g30160	1	0	0	0	1	1	0	2	5	1	0	0	0	0	0	0	0
Ma04_g31800	0	0	0	1	0	0	3	0	1	1	0	0	2	0	1	6	0
Ma04_g31880	0	0	2	0	0	0	0	1	0	1	1	0	1	1	2	0	1
Ma04_g32240	0	0	1	0	0	0	0	2	2	4	2	0	0	1	0	0	1
Ma04_g33920	3	7	2	2	4	1	1	0	1	1	0	0	1	1	1	3	1
Ma04_g34300	1	0	0	1	2	1	0	2	4	1	1	2	1	1	0	0	1
Ma04_g34660	0	1	0	0	0	0	0	1	0	0	0	3	0	0	0	0	0
Ma04_g35350	0	0	0	0	0	0	0	0	0	0	0	0	0	0	0	0	0
Ma04_g35730	0	0	4	0	0	0	0	0	1	0	0	0	0	0	0	0	0
Ma04_g35890	1	0	0	0	0	0	0	1	2	1	0	0	0	1	0	1	0
Ma04_g38740	0	0	1	0	0	0	0	0	0	0	0	0	1	1	1	0	1
Ma05_g01100	0	0	0	0	0	0	1	0	0	0	0	0	0	0	0	0	0
Ma05_g01880	0	0	2	1	0	0	0	2	3	2	0	2	2	1	1	2	2
Ma05_g03340	0	0	2	1	1	0	1	1	0	2	0	0	0	0	0	0	0
Ma05_g03690	10	0	1	14	2	25	28	1	0	1	1	1	2	0	8	1	0
Ma05_g05670	1	0	1	0	0	0	0	1	2	0	0	0	0	0	0	1	0
Ma05_g06310	4	1	1	4	4	4	8	1	3	1	1	0	0	0	1	0	0
Ma05_g07140	4	0	0	0	0	0	0	0	1	1	1	0	0	0	0	2	0
Ma05_g07450	4	0	0	2	4	3	1	1	2	1	1	0	0	0	0	0	0
Ma05_g08960	27	1	9	34	47	53	23	17	38	11	8	10	9	10	3	9	12
Ma05_g10430	0	0	2	1	0	0	0	2	1	1	2	3	3	6	2	1	4
Ma05_g12030	4	1	2	1	1	1	2	3	8	2	1	2	2	2	2	1	1
Ma05_g14510	1	0	4	48	0	1	190	1	0	1	2	0	1	1	1	3	0
Ma05_g17720	0	0	1	0	0	0	0	0	1	0	0	0	4	6	4	2	4
Ma05_g18420	0	0	0	0	0	1	0	1	4	0	0	0	0	0	0	0	0
Ma05_g18710	4	0	3	3	4	4	5	3	5	2	3	0	2	1	4	2	1
Ma05_g19630	0	0	6	0	0	0	0	1	2	3	0	1	1	1	0	2	0
Ma05_g20320	1	0	2	10	5	14	19	0	0	1	0	0	1	1	0	1	0
Ma05_g20740	0	4	1	2	9	0	0	2	5	1	2	0	1	0	2	1	0
Ma05_g20940	1	0	5	11	0	0	44	1	1	0	1	0	0	1	0	0	1
Ma05_g23480	0	0	0	0	0	0	0	2	3	5	0	0	0	0	0	0	1
Ma05_g23640	0	0	1	1	0	3	0	2	7	0	2	0	2	1	3	0	2
Ma05_g24200	0	0	0	0	0	0	0	0	1	0	0	0	0	0	0	0	0
Ma05_g24840	0	0	0	0	0	0	0	0	0	0	0	0	0	0	0	0	0
Ma05_g25150	0	0	6	2	0	0	0	3	4	4	5	1	3	3	2	2	4
Ma05_g25490	0	1	2	2	1	3	1	1	2	2	1	1	1	2	1	1	2
Ma05_g25630	0	0	0	4	15	0	1	1	6	0	0	0	0	0	0	0	0
Ma05_g25680	1	0	1	2	2	1	3	7	24	2	3	1	0	0	1	1	0
Ma05_g28370	0	0	4	4	9	4	1	1	0	1	0	1	2	2	1	1	2
Ma05_g30120	0	3	16	1	0	1	0	4	7	4	2	2	2	2	1	2	2
Ma05_g30720	0	0	19	1	0	0	0	7	12	10	2	4	3	3	3	1	6
Ma05_g31160	9	3	5	6	7	7	7	5	7	4	4	3	2	2	3	2	4
Ma05_g31440	0	3	2	68	266	2	0	43	12	88	71	2	26	2	89	9	2
Ma06_g00910	0	7	1	5	1	1	15	0	0	0	0	0	1	1	1	3	0
Ma06_g03570	0	0	4	2	0	0	0	3	5	3	3	4	5	7	4	3	7
Ma06_g04210	0	0	5	2	1	0	0	5	5	2	7	4	3	4	3	2	5
Ma06_g04240	1	0	1	1	4	0	0	7	29	0	0	0	0	0	0	0	0
Ma06_g04270	11	1	1	1	0	1	2	3	6	3	2	1	0	0	1	0	0
Ma06_g04370	7	0	10	13	5	29	15	1	3	0	0	0	2	1	6	1	0
Ma06_g05680	0	0	7	4	0	0	0	20	15	34	15	16	8	9	2	11	10
Ma06_g05960	0	0	6	4	4	0	0	13	9	19	13	9	4	4	3	9	2
Ma06_g06660	3	2	2	3	2	3	6	2	3	1	2	2	1	1	1	1	1
Ma06_g08100	3	0	0	0	0	0	0	0	0	0	0	0	0	0	0	0	0
Ma06_g08440	3	0	1	0	0	0	0	0	0	0	0	0	0	1	0	0	0
Ma06_g08910	4	3	5	28	68	23	16	8	6	11	9	5	10	4	20	14	3
Ma06_g11140	4	0	1	14	6	33	16	2	4	2	0	1	1	0	4	1	0
Ma06_g11270	0	1	10	0	0	0	0	0	1	0	0	0	1	0	1	3	0
Ma06_g12110	0	1	1	1	0	1	1	1	2	1	2	1	1	1	1	1	2
Ma06_g12160	1	1	1	1	1	2	2	1	2	0	1	1	1	0	0	2	1
Ma06_g14470	0	0	3	1	1	1	2	2	1	2	2	2	1	0	1	3	0
Ma06_g16350	0	1	0	3	4	2	5	1	2	1	1	2	1	0	0	1	0
Ma06_g16920	3	1	40	38	30	13	13	60	108	51	34	46	54	72	36	45	61
Ma06_g17440	0	0	0	0	0	0	0	1	1	1	0	1	1	0	1	1	2
Ma06_g19030	0	0	2	0	0	0	1	3	8	1	1	1	1	1	0	1	0
Ma06_g27210	2	2	8	6	2	6	16	3	4	5	0	2	2	2	1	2	2
Ma06_g29060	9	2	0	0	0	0	1	2	7	1	0	0	0	0	0	0	0
Ma06_g31020	7	3	7	6	3	9	8	1	2	2	2	0	2	1	7	1	1
Ma06_g32530	7	0	0	2	1	4	2	0	0	0	0	0	0	0	0	0	1
Ma06_g33100	4	6	3	3	2	3	3	4	5	3	2	6	3	4	2	2	5
Ma06_g33190	0	0	0	1	1	1	1	0	0	0	0	0	0	0	0	0	0
Ma06_g33430	0	1	11	2	1	1	0	8	20	7	2	4	3	5	3	4	3
Ma06_g33920	0	7	10	4	3	0	1	5	5	4	3	6	10	13	8	9	9
Ma06_g35430	6	15	28	31	49	14	31	20	37	22	13	8	33	24	63	25	17
Ma06_g35620	0	0	0	0	0	0	0	0	0	0	0	0	0	0	1	0	0
Ma06_g37660	0	0	0	0	0	0	0	0	0	0	0	0	0	0	0	0	0
Ma06_g38880	1	0	0	1	2	0	0	0	0	0	0	0	0	0	0	0	0
Ma07_g00270	0	0	1	1	1	0	0	0	1	0	0	0	0	1	0	0	0
Ma07_g02470	5	1	4	5	5	5	7	5	6	7	2	3	3	3	2	2	4
Ma07_g05660	0	0	0	0	0	0	0	0	0	0	0	0	1	0	0	2	0
Ma07_g05780	0	1	1	3	6	2	1	2	3	2	2	1	2	1	1	2	2
Ma07_g08110	0	1	5	1	0	0	4	0	1	0	0	0	0	0	0	1	0
Ma07_g10330	2	4	4	1	1	1	2	2	1	2	1	2	2	2	1	1	3
Ma07_g10340	2	11	6	23	82	3	2	11	22	10	8	5	14	8	15	28	5
Ma07_g11110	0	0	0	3	5	5	1	0	0	0	0	0	0	0	0	0	0
Ma07_g12330	0	0	1	7	5	18	3	1	2	1	0	0	0	0	1	0	0
Ma07_g13590	0	0	1	6	4	16	3	0	1	0	0	0	0	0	0	0	0
Ma07_g17600	1	1	8	2	4	0	1	1	1	1	0	1	2	2	2	2	2
Ma07_g19350	2	0	1	22	18	44	23	4	15	0	0	1	9	1	26	11	0
Ma07_g19470	3	0	15	7	5	6	6	54	80	62	33	42	10	14	6	9	10
Ma07_g19700	0	0	0	0	0	0	0	1	4	0	0	0	0	0	0	0	0
Ma07_g19720	0	0	7	7	26	1	0	5	11	6	3	1	4	2	12	1	1
Ma07_g19880*	1	1	5	4	0	0	2	10	14	7	11	8	9	12	3	8	12
Ma07_g19890*	0	0	0	0	0	0	0	0	2	0	0	0	0	0	0	0	0
Ma07_g20020	2	0	1	0	0	1	1	0	0	1	0	0	0	0	1	1	0
Ma07_g20990	0	0	2	0	0	0	0	2	2	1	3	0	2	2	1	1	1
Ma07_g22540	5	1	0	8	13	12	7	1	3	0	0	0	1	0	3	1	0
Ma07_g23060	0	1	2	1	2	0	0	2	4	2	3	1	1	2	1	1	1
Ma07_g23180	3	1	1	3	8	1	0	4	14	1	1	0	3	4	2	1	5
Ma07_g23230*	0	171	9	2	2	1	3	34	6	10	12	106	9	1	2	33	2
Ma07_g23240*	16	23	7	9	9	12	11	9	7	10	12	6	5	6	3	3	7
Ma07_g26530	0	1	1	1	0	2	1	1	4	0	0	0	0	0	0	0	0
Ma08_g01300	6	0	3	6	12	3	7	0	0	0	0	0	1	0	0	2	0
Ma08_g01760	6	0	0	2	2	2	4	0	1	0	0	0	1	0	1	1	1
Ma08_g02100	0	0	0	0	0	0	0	0	0	0	0	0	0	0	0	0	0
Ma08_g02450	1	3	5	2	2	1	3	7	7	10	9	1	5	3	13	2	3
Ma08_g03420	0	0	1	0	0	0	0	0	0	0	0	0	0	0	0	0	0
Ma08_g10260	1	0	3	7	16	3	1	4	12	1	1	3	13	18	6	7	19
Ma08_g10600	0	0	0	0	0	0	1	0	0	1	1	0	0	0	0	1	0
Ma08_g11120	0	0	0	0	0	0	0	0	1	1	0	0	0	0	0	0	0
Ma08_g12510	0	0	3	1	0	0	0	2	3	2	2	0	2	2	1	1	5
Ma08_g13070	0	0	10	4	0	0	0	17	12	30	18	8	15	19	7	12	21
Ma08_g14720	0	1	5	5	14	1	0	2	4	1	3	2	6	8	6	2	7
Ma08_g15820	0	1	4	2	1	1	2	6	7	6	6	4	4	5	4	5	2
Ma08_g15960	2	6	3	1	2	1	1	4	5	1	2	5	2	2	1	1	2
Ma08_g16760	2	0	2	10	5	13	21	3	5	3	0	3	3	1	4	7	0
Ma08_g17860	0	0	1	2	0	0	8	0	0	0	0	0	2	0	0	7	0
Ma08_g18540	0	0	0	1	0	1	2	0	0	0	0	0	0	0	0	2	0
Ma08_g23390	0	0	0	0	0	0	0	2	6	0	0	0	0	0	1	0	0
Ma08_g25570	7	1	16	11	7	6	7	20	20	26	25	10	19	16	19	16	23
Ma08_g25960	9	11	9	21	22	32	15	13	18	17	11	5	9	9	9	5	13
Ma08_g26720	1	0	7	9	19	4	13	1	1	2	1	1	1	0	1	1	1
Ma08_g30360	0	0	5	0	0	0	0	1	4	0	0	0	1	0	0	3	0
Ma08_g31720	0	0	1	1	1	0	0	0	0	0	1	0	0	0	0	0	0
Ma08_g32760	1	3	11	4	4	1	1	5	5	4	9	3	9	8	16	5	7
Ma08_g34230	0	0	2	1	0	1	0	2	4	1	1	1	2	1	1	2	2
Ma08_g34710	0	0	15	1	0	0	0	16	17	20	8	20	5	5	4	6	7
Ma09_g03310	2	1	11	4	8	3	1	2	5	1	1	2	3	3	5	1	3
Ma09_g03740	8	1	3	4	5	5	4	4	6	3	3	4	2	2	2	2	2
Ma09_g04930	0	2	12	2	0	0	0	10	7	22	7	3	5	5	9	4	3
Ma09_g06730	1	0	0	0	0	0	0	1	1	1	0	0	0	0	0	0	0
Ma09_g08140	0	0	2	0	1	0	0	3	10	0	0	0	0	0	0	0	0
Ma09_g08260	15	1	9	6	8	4	3	9	9	13	10	4	8	9	8	6	8
Ma09_g09400	0	0	7	1	0	0	1	1	2	2	0	1	1	0	1	1	0
Ma09_g09720	1	0	3	1	0	1	0	2	2	2	2	1	1	1	0	1	1
Ma09_g10800	0	0	5	0	0	0	0	1	1	2	0	2	1	1	0	1	0
Ma09_g11770	2	0	0	0	0	0	0	0	0	0	0	0	0	0	0	0	0
Ma09_g13170	0	0	5	3	0	0	0	6	7	4	10	4	9	12	4	5	13
Ma09_g14260	0	0	1	0	0	0	0	0	0	0	0	0	1	3	0	2	1
Ma09_g15050	1	0	0	0	0	0	0	1	0	0	1	1	1	1	0	0	0
Ma09_g15130	1	0	1	0	0	0	0	1	0	0	3	0	1	1	0	1	1
Ma09_g15440	0	0	1	2	5	1	0	5	9	3	2	4	1	1	2	2	1
Ma09_g15940	2	0	2	3	0	0	13	0	0	0	0	0	0	0	0	0	0
Ma09_g16940	3	0	0	4	5	8	2	0	1	0	0	0	1	2	1	0	1
Ma09_g16980	0	1	7	0	0	0	0	1	4	1	0	1	1	1	1	1	1
Ma09_g20280	0	0	62	8	0	0	0	44	31	73	45	27	26	30	21	29	23
Ma09_g22730	0	0	2	0	0	0	0	1	1	0	0	1	1	1	1	1	1
Ma09_g23100	32	2	3	36	45	65	32	10	9	21	9	1	5	2	12	4	3
Ma09_g24640	0	0	8	4	3	0	8	7	12	6	3	6	4	5	2	6	4
Ma09_g25010	4	0	2	4	3	4	7	1	1	1	2	0	2	1	2	1	2
Ma09_g25590	0	0	2	0	0	0	0	0	0	0	0	0	0	0	0	0	0
Ma09_g27990	0	0	1	1	2	0	0	1	2	2	0	1	2	2	1	1	3
Ma09_g28970	1	0	0	7	26	2	0	1	4	0	0	0	0	0	0	0	0
Ma09_g29010	0	2	32	6	0	1	3	25	26	32	15	25	15	16	7	17	22
Ma09_g29660	0	0	0	0	0	2	0	0	0	0	0	0	0	0	0	0	0
Ma10_g01730	0	0	0	1	4	1	0	0	0	0	0	0	0	0	0	0	0
Ma10_g01750	0	0	0	0	1	1	0	0	0	0	0	0	0	0	0	0	0
Ma10_g04420	0	0	1	4	10	2	2	1	2	1	1	0	1	1	3	0	0
Ma10_g04920	3	0	0	1	3	2	0	0	1	0	0	0	0	0	1	0	0
Ma10_g05260	0	0	0	1	1	1	3	0	0	0	0	0	0	0	0	0	0
Ma10_g05680	0	0	0	0	0	0	0	0	0	0	1	0	0	0	0	0	0
Ma10_g06140	0	0	0	0	0	0	1	0	1	0	0	0	0	0	0	0	0
Ma10_g09100	0	0	0	1	4	0	0	0	0	0	0	0	0	0	0	0	0
Ma10_g09370	0	0	4	3	1	0	5	2	2	3	3	1	3	3	2	4	2
Ma10_g10820	0	0	2	0	0	0	0	4	14	1	0	1	1	2	1	1	1
Ma10_g11100	0	1	6	2	0	0	6	0	0	0	0	0	2	0	0	7	0
Ma10_g13000	3	0	5	10	2	21	16	1	1	1	0	2	1	1	1	1	1
Ma10_g13640	2	1	40	31	30	34	12	22	25	19	20	25	35	48	21	28	44
Ma10_g14150	0	1	1	2	2	3	4	1	3	1	0	1	1	0	1	1	0
Ma10_g14950	0	0	3	2	0	0	0	4	8	1	2	3	4	4	2	3	7
Ma10_g16050	5	1	10	28	74	23	13	9	14	16	4	3	7	4	16	3	4
Ma10_g17650	0	30	0	0	0	0	0	4	13	1	0	0	2	2	2	4	1
Ma10_g18840	3	1	5	2	1	2	2	4	6	3	6	2	3	5	2	2	5
Ma10_g19130	0	0	2	0	0	0	0	1	0	0	1	1	1	0	1	0	1
Ma10_g19820	1	0	2	6	0	0	22	0	0	0	0	0	5	0	12	9	0
Ma10_g19970	1	0	0	2	6	3	1	1	3	1	0	0	0	0	0	0	0
Ma10_g24510	9	4	2	7	16	6	4	3	4	2	4	2	1	1	1	1	2
Ma10_g25660	1	0	0	0	0	0	0	1	1	0	0	1	0	0	0	0	0
Ma10_g26540	5	7	6	4	3	4	4	6	8	4	4	6	4	5	2	3	6
Ma10_g26660	2	0	6	3	1	2	2	10	14	11	8	5	4	3	3	5	6
Ma10_g29230	6	0	1	4	6	9	3	0	1	1	0	0	1	0	2	0	0
Ma10_g29290	4	1	1	2	2	2	2	1	1	0	1	1	1	1	0	0	1
Ma10_g29660	2	0	1	15	60	0	0	1	3	0	0	0	0	0	0	0	0
Ma10_g29900	5	1	9	10	7	11	11	8	8	7	9	8	9	11	10	10	7
Ma11_g00330	0	0	4	0	0	0	0	0	1	0	0	1	0	0	0	0	0
Ma11_g00350	0	0	6	5	13	1	0	3	5	2	2	2	5	7	5	3	6
Ma11_g02310	0	0	2	1	0	0	0	2	1	1	4	0	2	2	1	1	4
Ma11_g03860	0	0	2	13	47	1	0	5	14	4	1	0	2	1	5	1	1
Ma11_g04680	20	2	0	32	6	57	64	0	1	1	0	0	4	0	7	10	0
Ma11_g06880	22	4	15	13	7	19	12	31	41	39	30	14	7	6	4	9	11
Ma11_g07330	0	0	0	0	0	0	0	0	0	0	0	0	0	0	0	0	0
Ma11_g07530	0	0	5	2	1	1	1	6	3	14	4	2	3	5	1	4	2
Ma11_g08730	1	1	1	1	1	1	1	1	1	1	0	1	1	0	1	0	1
Ma11_g10680	1	2	5	2	1	0	3	12	21	20	6	3	6	7	5	8	6
Ma11_g10710	0	1	2	1	4	0	0	2	5	2	1	2	3	1	6	2	1
Ma11_g11300	2	0	0	0	0	0	1	0	0	0	0	0	0	0	0	1	0
Ma11_g11940	0	0	5	0	0	0	0	0	1	0	0	0	0	0	0	0	0
Ma11_g14670	0	1	2	0	0	0	0	0	0	0	0	0	2	0	2	5	0
Ma11_g15740	4	0	2	5	7	4	6	6	9	4	6	5	2	1	3	2	1
Ma11_g16150	1	2	3	10	37	2	1	4	7	4	6	1	3	3	3	2	4
Ma11_g16430	0	0	0	0	0	0	0	0	0	0	0	0	0	0	0	0	0
Ma11_g19220	1	0	1	0	0	0	0	0	1	1	0	0	2	0	0	7	0
Ma11_g21160	1	0	9	3	0	1	12	2	4	0	4	0	0	1	0	0	0
Ma11_g21730	0	0	2	1	0	1	0	2	2	3	0	1	2	4	0	2	2
Ma11_g21820	0	0	0	2	2	4	0	0	0	0	0	0	0	0	1	0	0
Ma11_g23010	0	0	6	1	0	0	0	3	3	5	2	2	2	3	1	2	3
Ma11_g23420	1	0	2	0	0	0	0	1	2	0	0	0	0	0	0	0	0

The intensity of green background is correlated to expression level given in FPKM.

* indicate paralogs.

Our expression analyses revealed that *M*. *acuminata* MYBs have diverse expression patterns in different organs. Many of the *MaMYBs* exhibited low transcript abundance levels with expression in only one or a few organs. This is consistent with other transcription factor genes, typically found to be expressed in this manner due to functional specificity and diversity. The highest number of expressed *MaMYB* genes (221; 75.4%) is observed in roots, followed by pulp (209; 71.3%), leaf (203; 69.3%), peel (196; 66.9%) and embryonic cell suspension (129; 44%). The fewest *MaMYB* genes are expressed in seedlings (98; 33.4%) in the considered dataset. 40 *MaMYB* genes (13.7%) were expressed in all samples analysed (albeit with varying expression levels), which suggested that these MaMYBs play regulatory roles at multiple developmental stages in multiple tissues. 13 *MaMYB* genes (4.4%) lacked expression information in any of the analysed samples, possibly indicating that these genes are expressed in other organs (e.g. pseudostem, flower, bract), specific cells, at specific developmental stages, under special conditions or are pseudogenes. 280 MaMYBs (95.6%) are expressed in at least one analysed sample, although the transcript abundance of some genes was very low. Some *MaMYB* genes were expressed in all analysed RNA-Seq samples at similar levels (e.g. R2R3-MYBs *Ma06_g33440* and *Ma11_g06880*) while others show variance in transcript abundance with low (no) levels in one or several organs and high levels in others (or vice versa). For example, *Ma02_g16570*, *Ma03_g09310*, *Ma07_g11110*, *Ma10_g01730*, *Ma10_g05260* and *Ma10_g09100* show organ-specific expression, as their transcripts were exclusively detected in leaves, which hints to leaf-specific functionality. *Ma03_g07840* and *Ma07_g19470* were found to be predominantly expressed in pulp, showing expression also in other analysed organs, but not in the seedling. Overall, these results suggests that the corresponding MaMYB regulators are limited to distinct organs, tissues, cells or conditions.

Some paralogous *MaMYB* genes clustered in the genome (Tables 1 and 2) showed different expression profiles, while other clustered paralogous *MaMYB* genes did not. For example, the clustered *R2R3-MYB* genes *Ma07_g19880* and *Ma07_g19890* (both in the proanthocyanidin-related cluster 25): while *Ma07_g19880* is expressed in pulps, peels, roots, and leaves, *Ma07_g19890* is nearly not expressed in the analysed organs. The expression pattern of *Ma03_g07840* and *Ma03_g07850* (also both in the proanthocyanidin-related cluster 25) is, in contrast, very similar, with low expression in root, leaf, pulp and peel, but no expression in embyonic cells and roots. These results could point to functional redundancy of the genes *Ma03_g07840* and *Ma03_g07850*, while *Ma07_g19880* and *Ma07_g19890* could be (partly) involved in distinct, tissue-specific aspects of specialised mebabolite biosynthesis. The functional categorisation and its expression domains make *Ma07_g19880* a good candidate to encode a proanthocyanidin biosynthesis regulator in developing banana fruits (pulp and peel), and thus an excellent target for genetic manipulation of proanthocyanidin content, known to influence senescence of banana fruit [46], to maintain the freshness of harvested banana fruit.

## Conclusions

The present genome-wide identification, chromosomal organisation, functional classification and expression analyses of *M*. *acuminata MYB* genes provide a first step towards cloning and functional dissection to decode the role of *MYB* genes in this economically interesting species. Further, knowledge of selected *MYB* genes can be biotechnologically utilized for improvement of fruit quality, yield, disease resistance, tolerance to biotic and abiotic stresses, and the biosynthesis of pharmaceutical compounds.

## Supporting information

S1 Table MYB domain consensus sequence of R2R3-MYBs, used for tBLASTn searches. (DOCX)

S2 Table RNA-Seq raw data used for expression analysis. (DOCX)

S1 File Multi-FASTA file with *MaMYB* CDS sequences. (FASTA)

S2 File Multi-FASTA file with *MaMYB* peptide sequences. (FASTA)

S3 File *MaMYB* genes General Feature Format (GFF) file. For use in genome viewers/browsers on the *M*. *accuminata* pseudochromosomes.
(GFF)
