# Supplementary material for: The R2R3-MYB gene family in banana (Musa acuminata): Genome-wide identification, classification and expression patterns
Source: PLoS One. 2020 Oct 6;15(10):e0239275. doi: 10.1371/journal.pone.0239275 (PMC7537896; doi:10.1371/journal.pone.0239275)
Supplement: S1 Table — (DOCX) [file pone.0239275.s001.docx]

**S1 Table. MYB domain consensus sequence of R2R3-MYBs, used for tBLASTn searches.**

GxWxxxEDxxLxxxxxxxGxxxWxxxxxxxGLxRxxKSCRLRWxNYLxPxxxxGxxxxxExxxxxxLxxxxGNxWxxIAxxxPxRTxNxxKNxW
